# Hybrid Cells Derived from Human Breast Cancer Cells and Human Breast Epithelial Cells Exhibit Differential TLR4 and TLR9 Signaling

**DOI:** 10.3390/ijms17050726

**Published:** 2016-05-13

**Authors:** Songül Tosun, Sabrina Fried, Bernd Niggemann, Kurt S. Zänker, Thomas Dittmar

**Affiliations:** 1 Institute of Immunology & Experimental Oncology, Center for Biomedical Education and Research (ZBAF), University of Witten/Herdecke, Stockumer Str. 10, 58448 Witten, Germany; songuel.tosun@uni-wh.de (S.T.); sabrina_fried@gmx.de (S.F.); bernd.niggemann@uni-wh.de (B.N.); kurt.zaenker@uni-wh.de (K.S.Z.); 2 Faculty of Medicine, Ruhr University Bochum, Universitätsstraße 150, 44801 Bochum, Germany

**Keywords:** cell fusion, breast cancer, *TLR4*, *TLR9*, signal transduction

## Abstract

*TLRs* are important receptors of cells of the innate immune system since they recognize various structurally conserved molecular patterns of different pathogens as well as endogenous ligands. In cancer, the role of *TLRs* is still controversial due to findings that both regression and progression of tumors could depend on TLR signaling. In the present study, M13SV1-EGFP-Neo human breast epithelial cells, MDA-MB-435-Hyg human breast cancer cells and two hybrids M13MDA435-1 and -3 were investigated for *TLR4* and *TLR9* expression and signaling. RT-PCR data revealed that LPS and CpG-ODN induced the expression of pro-inflammatory cytokines, like *IFN-*β, *TNF-*α, *IL-1*β and *IL-6* in hybrid cells, but not parental cells. Interestingly, validation of RT-PCR data by Western blot showed detectable protein levels solely after LPS stimulation, suggesting that regulatory mechanisms are also controlled by TLR signaling. Analysis of *pAKT* and *pERK1/2* levels upon LPS and CpG-ODN stimulation revealed a differential phosphorylation pattern in all cells. Finally, the migratory behavior of the cells was investigated showing that both LPS and CpG-ODN potently blocked the locomotory activity of the hybrid cells in a dose-dependent manner. In summary, hybrid cells exhibit differential *TLR4* and *TLR9* signaling.

## 1. Introduction

*TLRs* are type I transmembrane receptors that belong to the innate immune system [1,2,3]. They are chiefly expressed by immune competent cells, like macrophages, dendritic cells, B- and T-lymphocytes, and they do recognize structurally conserved pathogen derived molecules, so-called PAMPs [1,2,3]. To date, 10 different *TLRs* have been identified in humans so far, each possessing a specificity for a certain ligand or ligands. For instance, *TLR4* recognizes bacterial lipopolysaccharides and *TLR5* bacterial flagella, whereas *TLR9* binds unmethylated CpG-DNA of bacterial origin [1,2,3]. However, within the past few years it has become evident that *TLRs* also recognize endogenous ligands, also named DAMPs, such as extracellular matrix components (*TLR4*), heat shock proteins (*TLR2/4*), HMGB1 (*TLR4*) and self DNA (*TLR9*) [2]. Usually, the recognition of DAMPs plays a crucial role in identifying and removing cell debris and inducing wound healing in response to non-pathogen-induced tissue damage [4]. Due to recognizing intracellular proteins to be released and presented by necrotic, apoptotic and/or necroptotic cells, DAMPs and its receptors play a crucial role in chronic inflammatory diseases including rheumatoid arthritis [5] and even cancer [6]. It is well recognized that the tumor microenvironment resembles chronically inflamed tissue and thus cancer has been called “wounds that do not heal” [7].

However, the role of *TLRs* in cancer is much debated due to contradictory reports. Some studies provided evidence that *TLR* ligands, such as LPS from Gram negative bacteria or CpG-ODN, might be efficacious in the treatment of various cancer types, including colorectal cancer, glioblastoma, hepatocellular carcinoma and myeloma [8,9,10,11,12]. On the contrary, several reports demonstrated that TLR expression of cancer cells might be rather associated with tumor progression. For instance, LPS could induce epithelial-to-mesenchymal transition (EMT) in cancer cells and has been associated with cancer cell invasion and metastasis in a *TLR4* dependent manner [13,14,15]. Moreover, *TLR4* expression in breast cancer and ovarian cancer has been correlated to paclitaxel chemoresistance [16,17]. Likewise, *TLR9* agonists like CpG-ODN or even DNA from dead cells could promote cancer cell invasion [18,19,20]. *TLR9* has also been suggested as a prognostic factor in breast cancer, whereas Tuomela *et al.* demonstrated that rather low *TLR9* levels define an aggressive subtype of triple-negative breast cancer [21].

Cell fusion has been suggested as a driving force in cancer progression because a plethora of data provided evidence that hybrid cells derived from tumor cells and tumor cells or tumor cells and normal cells, like macrophages [22,23] or epithelial cells [24,25,26], could exhibit novel properties, such as an enhanced drug resistance or an increased metastatic activity (for review see: [27,28,29,30,31]). We thus investigated M13MDA435-1 and -3 hybrid cells in comparison to their parental cells (human M13SV1-EGFP-Neo breast epithelial cells and human MDA-MB-435-Hyg breast cancer cells) [25,32] for *TLR* expression and signaling. We have recently demonstrated that LPS potently induced apoptosis in M13MDA435 hybrid cell clones, but not in parental cells [33]. Here, we additionally investigated the cells for *TLR9* expression and signaling.

## 2. Results

### 2.1. M13MDA435 Hybrid Cells Respond Differently to CpG-ODN and LPS Stimulation

**Figure 1:**
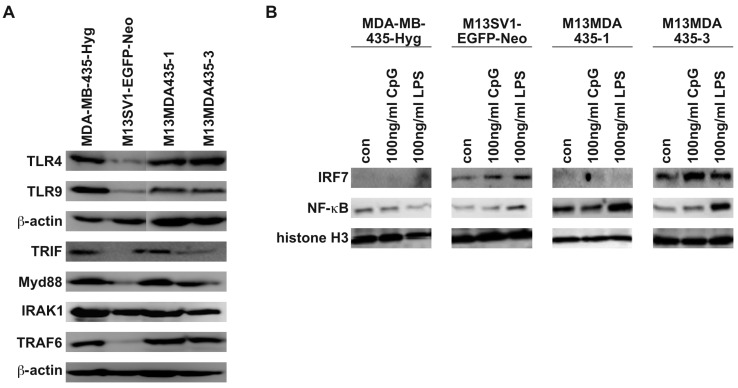
Expression of *TLR4* and *TLR9* and components of the *TLR* signal transduction cascade. (**A**) M13SV1-EGFP-Neo breast epithelial cells express lower levels of *TLR4*, *TLR9*, *TRIF*, *Myd88* and *TRAF6* in comparison to the other cells; (**B**) Nuclear translocation of *IRF7* was found in LPS and CpG-ODN (CpG) treated M13SV1-EGFP-neo cells and M13MDA435-3 hybrid cells, but not MDA-MB-435-Hyg breast cancer cells and M13MDA435-1 hybrid cells. By contrast, nuclear translocation of *NF-*κ*B* was solely detected in LPS treated hybrid cells. Shown are representative Western blots of at least three independent experiments.

In accordance to recently published data MDA-MB-435-Hyg human breast cancer cells and M13MDA435-1 and -3 hybrid cells exhibited comparable expression levels of *TLR4*, *TLR9*, *TRIF*, *Myd88*, and *TRAF6* (Figure 1A). In contrast to this, the expression of these proteins was rather moderate to low in M13SV1-EGFP-Neo human breast epithelial cells (Figure 1A). However, all cell lines showed comparable *IRAK1* expression levels (Figure 1A). Stimulation of cells with either 100 ng/mL CpG-ODN and 100 ng/mL LPS, respectively, revealed a differential *IRF7* and *NF-*κ*B* activation. In accordance with recently published data [33], LPS treatment resulted in *NF-*κ*B* activation in both hybrid cells, but not in parental cells (Figure 1B). On the contrary, stimulation of cells with 100 ng/mL CpG-ODN did not activate *NF-*κ*B* signaling in all cell lines (Figure 1B). Analysis of *IRF7* activation upon CpG-ODN and LPS stimulation indicated that the cells responded differently. While in M13SV1-EGFP-Neo cells and M13MDA435-3 hybrid cells both CpG-ODN and LPS stimulation resulted in *IRF7* activation, no nuclear translocation of this transcription factor was detected in MDA-MB-435-Hyg breast cancer cells and M13MDA435-1 hybrid cells (Figure 1B).

### 2.2. M13MDA435 Hybrid Cells and Parental Cells Respond Differently to LPS and CpG-ODN Stimulation

Next, the expression of *NF-*κ*B* and *IRF7* target genes was investigated by RT-PCR and Western blot in MDA-MB-435-Hyg human breast cancer cells, M13SV1-EGFP-Neo human breast epithelial cells and M13MDA435-1 and -3 hybrid cells that were stimulated with LPS and CpG-ODN for 2, 6, 12, 24 and 48 h.

**Figure 2:**
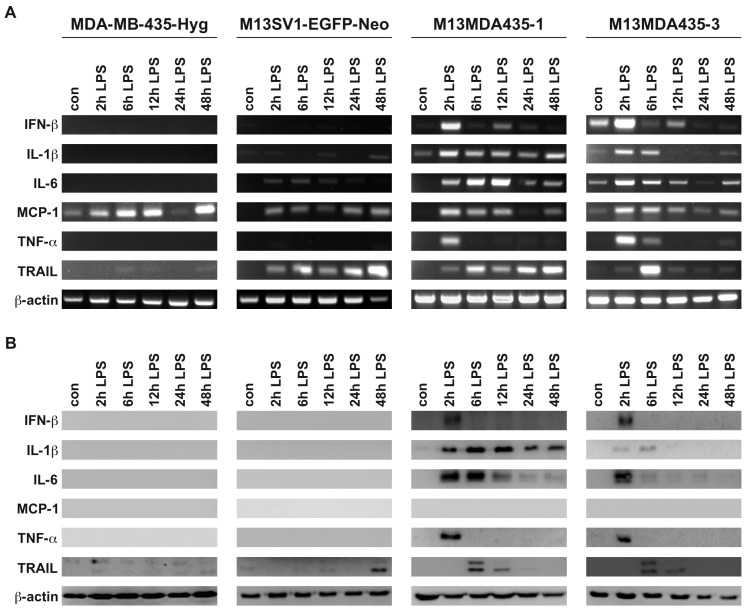
RT-PCR and Western blot analysis of target genes expressed in response to LPS stimulation. (**A**) RT-PCR data (**B**) Western blot data. All cell lines were treated with 100 ng/mL LPS for up to 48 h. Results show that the cell lines responded uniquely to LPS stimulation, which particularly applies for protein translation. Whereas in M13MDA435-1 and -3 hybrid cells most of the transcribed genes are expressed as proteins, protein expression is lacking in parental cells, which is putatively attributed to miRNA or lncRNA dependent mechanisms. Shown are representative RT-PCR and Western Blot data of three independent experiments.

We recently demonstrated that LPS stimulation lead to the induction of *TNF-*α and *IFN-*β in M13MDA435-1 and -3 hybrid cells, but not parental cells [33], which could be reproduced in this study (Figure 2). Even though both hybrid cell lines responded similarly in the overall expression of target genes in response to LPS stimulation, they differed markedly in the kinetics of gene/protein expression (Figure 2). For instance, a permanent LPS induced expression of *IL-1*β was detected in M13MDA435-1 hybrid cells on both mRNA and protein, whereas in M13MDA435-3 hybrid cells, *IL-1*β expression was solely detected after 2 to 6 h of LPS treatment (Figure 2). A marked expression of *TNF-*α was detected in both hybrid cell lines after 2 h of LPS stimulation (Figure 2). On the contrary, RT-PCR data showed a sustained of *IFN-*β expression in both hybrid cell lines after LPS stimulation with a first peak maximum after 2 h and a second one after 12 h (Figure 2A). However, *IFN-*β protein expression in dependence of LPS was solely detected after 2 h in the hybrid cells (Figure 2B). Differential mRNA and protein levels were further observed for *IL-6*, *MCP-1* and *TRAIL* in both hybrid cell lines. For instance, rather sustained *IL-6* and *TRAIL* mRNA levels in response to LPS stimulation were detected in M13MDA435-1 hybrid cells, whereas the peak maximum of *IL-6* protein expression was observed between 2 h and 6 h (Figure 2B). Prolonged stimulation of M13MDA435-1 hybrid cells with LPS was correlated with a decreasing *IL-6* protein expression (Figure 2B). Likewise, *TRAIL* protein expression was solely observed between 6 h to 12 h in LPS treated M13MDA453-1 hybrid cells (Figure 2B). 

RT-PCR analysis revealed that both parental cells responded differentially to LPS stimulation with the expression of appropriate target genes. In MDA-MB-435-Hyg breast cancer cells increased mRNA levels of *MCP-1* and *TRAIL* were observed after LPS stimulation (Figure 2A). Increased mRNA levels of *IL-6*, *MCP-1* and *TRAIL* were observed in LPS treated M13SV1-EGFP-Neo breast epithelial cells (Figure 2A) indicating that these cells exhibit a functional *TLR4* signaling despite rather low expression levels of *TLR4* and components of the *TLR4* signal transduction cascade (Figure 1A). In accordance to previously published data [33], neither *TNF-*α nor *IFN-*β expression was induced in LPS treated parental cell lines. However, in contrast to M13MDA435-1 and -3 hybrid cells, virtually no target gene protein expression was observed in LPS stimulated parental cells (Figure 2B). Only a slight *TRAIL* expression was detected in M13SV1-EGFP-Neo breast epithelial cells treated for 48 h with LPS (Figure 2B).

It is well recognized that the expression of proteins is also controlled by miRNAs or lncRNAs, which either block expression by degrading the mRNA or by blocking translation at the ribosome [34]. We thus conclude that the non-expression of target genes despite detectable mRNA levels might be attributed to such as a regulatory mechanism. This may particularly apply for MCP-1. Increased *MCP-1* mRNA levels in response to LPS stimulation were found in all investigated cell lines, whereas no *MCP-1* protein expression was observed (Figure 2).

**Figure 3:**
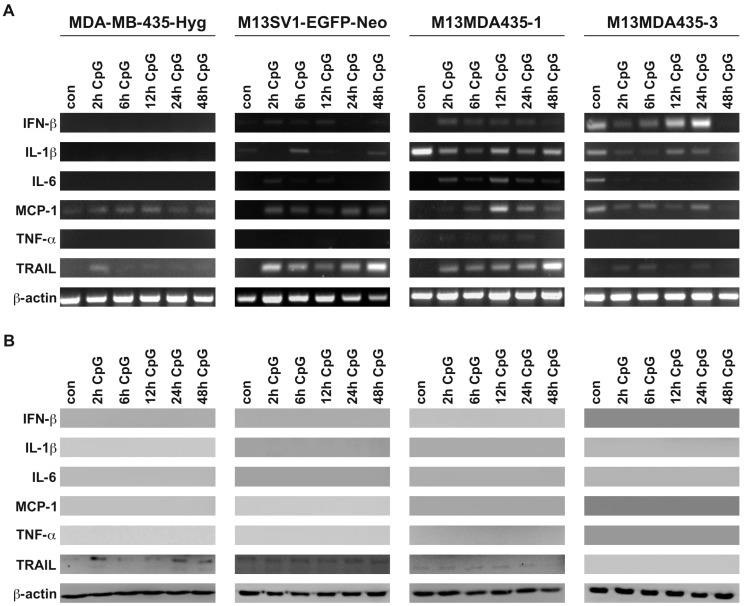
RT-PCR and Western blot analysis of target genes expressed in response to CpG-ODN stimulation. (**A**) RT-PCR data (**B**) Western blot data. All cell lines were treated with 100 ng/mL CpG-ODN for up to 48 h and exhibit a functional *TLR9* signaling as indicated by induction of target gene expression. Interestingly, only *TRAIL* protein expression in response to CpG-ODN stimulation was detected in parental cell lines and M13MDA435-1 hybrid cells, suggesting that the translation of other analyzed target genes is impaired by miRNA or lncRNA dependent mechanisms. Shown are representative RT-PCR and Western blot data of three independent experiments.

Treatment of cells with CpG-ODN was markedly different from LPS stimulation. Albeit CpG-ODN stimulation resulted in increased target gene mRNA levels in both hybrid cell lines and the M13SV1-EGFP-Neo breast epithelial cell line (Figure 3A) all analyzed target genes, with exception of *TRAIL*, were not expressed on a protein level (Figure 3B). On the one hand, these data indicate that the cells exhibit functional *TLR9* signaling, but at present it remains unknown why a protein translation did not occur in CpG-ODN-treated cells. As mentioned above, we conclude that most likely miRNA or lncRNA based regulatory processes are responsible for this observation.

### 2.3. Expression of Pro-Inflammatory and Apoptosis-Inducing Cytokines in Hybrid Cells Does Not Correlate with BAX and BCL-2 Expression Levels

**Figure 4:**
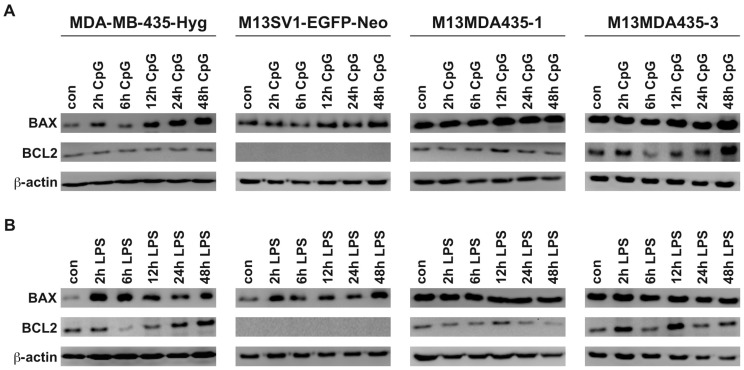
Expression of *BAX* and *BCL2* in dependence of CpG and LPS stimulation in the investigated cell lines. (**A**) Data for CpG-ODN (CpG) treated cells; (**B**) data for LPS treated cells. Interestingly, M13SV1-EGFP-Neo breast epithelial cells lack *BCL2* expression. Shown are representative data of at least three independent experiments.

We have recently demonstrated that LPS potently induced apoptosis in M13MDA435-1 and -3 hybrid cells in an *IFN-*β dependent manner [33]. Because of that *BAX* and *BCL-2* protein levels were determined by Western Blot of both CpG-ODN and LPS treated cells (Figure 4). The pro-apoptotic acting protein *BAX* was induced by both CpG-ODN and LPS in MDA-MB-435-Hyg breast cancer cells, whereas only LPS stimulation caused increased *BAX* levels in M13SV1-EGFP-Neo breast epithelial cells (Figure 4A,B). Interestingly, M13SV1-EGFP-Neo breast epithelial cells lack *BCL-2* expression (Figure 4A,B). In contrast to parental cells BAX expression levels remained unaltered in both CpG-ODN and LPS treated M13MDA435-1 and -3 hybrid cell clones (Figure 4A,B). Likewise, a slight to moderate up-regulation of *BCL-2* was observed in LPS treated hybrid cells (Figure 4A,B). This, however, is contrary to our previously published data showing that LPS potently induced apoptosis in M13MDA435-1 and -3 hybrid cells, but not parental cells [33]. It is well recognized that *BCL-2* belongs to the group of anti-apoptotic proteins [35].

### 2.4. CpG-ODN and LPS Both Induce AKT and ERK1/2 Signaling in Parental Cells and Hybrid Cells

**Figure 5:**
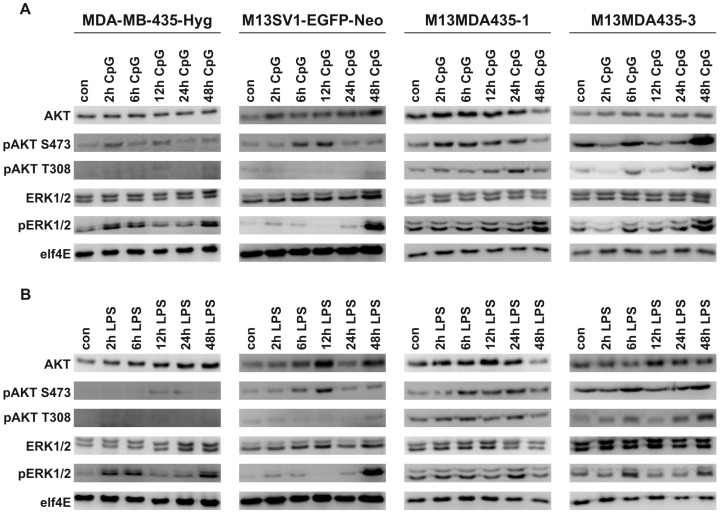
Activation of *AKT* and *ERK1/2* in dependence of CpG and LPS stimulation. Cells were stimulated with either 100 ng/mL CpG (**A**) or 100 ng/mL LPS (**B**), respectively. Shown are representative data of at least three independent experiments.

It is well recognized that stimulation of *TLR4* and *TLR9* also result in the engagement of *MAPK* signaling via *TRAF6* mediated activation on *TAB2*/*TAB3*/*TAK1* [3]. Conjointly, Bauerfeld *et al.* demonstrated that *TLR4* signaling could also activate *AKT* via a *Myd88*/*TRIF* dependent mechanism [36]. Similar findings were reported for *TLR9* signaling [37]. We thus investigated whether stimulation of MDA-MB-435-Hyg human breast cancer cells, M13SV1-EGFP-Neo human breast epithelial cells and their hybrids resulted in activation of *AKT* and *ERK1/2* signaling. In fact, increased *pAKT* (S473 and T308) and *pERK1/2* levels were detected in all cell lines upon CpG-ODN and LPS stimulation (Figure 5A,B), whereby the analyzed cell lines differed markedly in the kinetics of *AKT* and *ERK1/2* phosphorylation. In parental cells, both CpG and LPS resulted in increased *pAKT* S473 levels, but not *pAKT* T308 levels (Figure 5A,B). On the contrary, increased *pAKT* S473 and *pAKT* T308 levels were detected in both hybrid cell lines stimulated with CpG and LPS (Figure 5A,B). Phosphorylation of *AKT* at positions T308 and S473 is regulated via different signal transduction pathways [38]. While phosphorylation of the activation loop of *AKT* at T308 is facilitated by *PDK1* in a *PI3K* dependent manner, phosphorylation of *AKT* on the hydrophobic motif S473 is mediated by *mTORC2* [38]. Thus, two different *AKT* activating pathways are engaged in hybrid cells by *TLR4* and *TLR9* signaling. Interestingly, *pAKT* S473 levels were rather low to moderate in MDA-MB-435-Hyg human breast cancer cells, whereas in M13SV1-EGFP-Neo human breast epithelial cells higher *pAKT* S473 levels were detected with a maximum after 6 and 12 h (Figure 5A,B). On the contrary, markedly higher *pAKT* S473 levels were detected in M13MDA435-1 and -3 hybrid cells. Comparison of the kinetics of T308 and S473 *AKT* phosphorylation in M13MDA435-1 and -3 hybrid cells revealed a rather identical phosphorylation pattern in M13MDA435-3 hybrid cells (Figure 5A,B). Here, markedly increased *pAKT* T308 and S473 levels were detected after 6 and 24 h of CpG-ODN and LPS stimulation (Figure 5A,B). On the contrary, in CpG treated M13MDA435-1 hybrid cells highest *pAKT* S473 were observed after 2 to 6 h, whereas the maximum peak level of *pAKT* T308 was observed after 24 h (Figure 5A). Both parental cell lines showed a similar *ERK1/2* phosphorylation in response to both CpG-ODN and LPS. Increased *pERK1/2* were observed after 2, 6 and 48 h of stimulation with either CpG-ODN and LPS, respectively (Figure 5A,B). On the contrary, both hybrid cell clones exhibited a unique *ERK1/2* activation profile in response to CpG-ODN and LPS stimulation. For instance, a CpG-ODN dependent *ERK1/2* phosphorylation was first detected after 48 h in M13MDA435-1 hybrids, whereas in LPS treated cells *pERK1/2* were observed after 24 h (Figure 5A,B). On the contrary, *ERK1/2* phosphorylation in response to CpG-ODN stimulation was detected after 6, 12, 24 and 48 h in M13MDA435-3 hybrid cells (Figure 5A). Likewise, increased LPS-mediated *pERK1/2* levels were found after 6 and 48 h (Figure 5B). These data show that hybrid cell clones exhibit unique kinetics of *AKT* and *ERK1/2* activation in response to CpG-ODN and LPS stimulation.

### 2.5. The Migratory Activity of Parental Cells and Hybrid Cells is Impaired by CpG-ODN in a Dose-Dependent Manner

**Figure 6:**
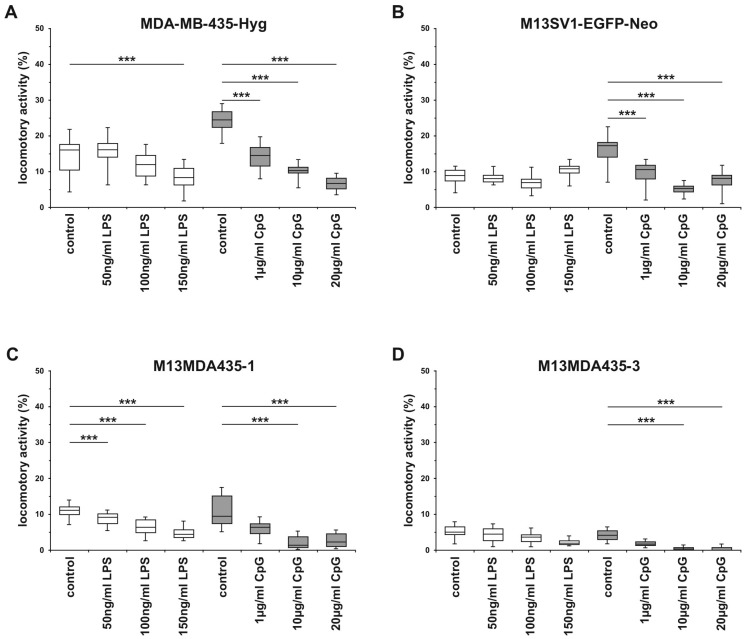
Cell migration data. (**A**) MDA-MB-435-Hyg human breast cancer cells; (**B**) M13SV1-EGFP-Neo human breast epithelial cells; (**C**) M13MDA435-1 hybrid cells; (**D**) M13MDA435-3 hybrid cells. Cells were stimulated with either LPS or CpG-ODN (CpG). CpG inhibited the migratory activity of all cells in a dose dependent manner. The mean locomotory activity ± S.E.M. of the cells is displayed as BoxPlots. Statistical Significance was calculated using the Mann-Whitney-*U*-test (*** <0.01). Shown are the means of at least three independent experiments.

*TLR4* and *TLR9* signaling have been associated with breast cancer progression due to findings revealing that both LPS and CpG-ODN could promote breast cancer cell migration, invasion and metastatic spreading [13,15,20]. We thus analyzed the cells locomotory activity within a 3D collagen matrix in dependence of different CpG-ODN and LPS concentrations. However, in contrast to studies providing evidence that LPS and CpG-ODN could foster breast cancer cell migration our migration data rather indicated an inhibitory effect of both compounds on the cells motility. The migratory activities of MDA-MB-435-Hyg human breast cancer cells and M13MDA435-1 and -3 hybrid cells were impaired by LPS in a dose dependent manner, whereas the migratory activity of M13SV1-EGFP-Neo breast epithelial cells remained unaffected in the presence of LPS (Figure 6). On the contrary, CpG-ODN inhibited the migratory activity of all investigated cell lines in a dose-dependent manner (Figure 6). As shown recently, LPS potently induced apoptosis in M13MDA4351 and -3 hybrid cells, but not parental cells [33], suggesting that the decreased migratory activity of hybrid cells within the presence of LPS might be attributed to an increased number of apoptotic cells. However, the viability of MDA-MB-435-Hyg cells was not affected by LPS, which also applies to CpG-ODN for all cell lines. Thus, the means by which LPS and CpG-ODN impairs the migration of the cells remains to be elucidated.

## 3. Discussion

In the present study, human M13SV1-EGFP-Neo breast epithelial cells, human MDA-MB-435-Hyg and two of their hybrids M13MDA435-1 and -3 [25,32] were analyzed for *TLR4* and *TLR9* expression and signaling. Our data show that although all cell lines express both receptors, they differed markedly in the kinetics of *TLR* specific signal transduction cascades and target gene expression. On the contrary, stimulation of *TLR4* or *TLR9* signaling with LPS or CpG-ODN, respectively, resulted in a decreased locomotory activity of all investigated cells.

The finding that LPS stimulation resulted in a time-dependent expression of LPS target genes, like *IL-1*β, *IL-6* and *TRAIL* in M13MDA435 hybrid cells, but not parental cells, is in line with previously published data [33]. Of interest in this context is the correlation of RT-PCR data and Western Blot data revealing marked differences. For instance, LPS-induced and sustained *TRAIL* mRNA levels were detectable in M13MDA435-1 hybrid cells for up to 48 h, whereas a maximum of *TRAIL* expression on a protein level was observed between 6 and 12 h (Figure 2B and Figure 3B). Similar findings were found for LPS-induced *TRAIL* expression in M13SV1-EGFP-Neo cells. Here, an increased *TRAIL* expression was found upon 48 h of LPS stimulation while *TRAIL* mRNA levels were already detected after 2 h of LPS stimulation (Figure 2B and Figure 3B). Likewise, MCP-1 expression was solely detected by RT-PCR, but not Western blot analysis (Figure 2B and Figure 3B), which particularly applies for *IFN-*β, *IL-1*β, and *IL-6* expression in CpG-ODN treated hybrid cells (Figure 2B and Figure 3B). These findings indicate that LPS and CpG-ODN not only induce target gene expression by activation of transcription factors, but most likely also by modulating the expression or the activation state of regulating molecules, like miRNA or lncRNA. Both, miRNAs and lncRNAs are well-known modulators of gene expression [39,40].

The finding that LPS potently induced apoptosis in M13MDA435-1 and -3 hybrid cells via an *IFN-*β dependent mechanism [33] still remains ambiguous, which also applies for the role of LPS and *TLR4* signaling in breast cancer. As mentioned in the introduction, *TLRs* do not only recognize PAMPs, but also DAMPs, which are derived from necrotic, apoptotic and necroptotic cells [4]. It would thus be of interest to study the impact of endogenous *TLR4* ligands, like extracellular matrix components or heat shock proteins, on parental cells and hybrid cells and whether they do also induce a differential *TLR4* signaling concomitant with a differential expression of specific target genes and induction of apoptosis in hybrid cells as compared to parental cells. In this context, it would be of interest to investigate whether necrotic, apoptotic and necroptotic cell-derived DAMPs could stimulate cells in a paracrine manner.

Stimulation of M13MDA435-1 and -3 hybrid cells with LPS resulted in the expression of pro-inflammatory cytokines including *IL-1*β, *TNF-*α, and *IL-6*. Whether the expression levels of these cytokines would be sufficient to activate macrophages or to direct macrophage differentiation towards a “M1-macrophage” or “classical activated macrophage” phenotype [4,41] remains to be elucidated. Tumor-associated macrophages have been identified as a double-edged sword in cancer progression [42]. “M1-macrophage” or “classical activated macrophage”, which are commonly present in acute inflammatory conditions [4,41,43,44], have been generally associated with “tumor rejection” and thus a better prognosis [41,45]. On the contrary, “M2-macrophages” or “wound-healing/resolution macrophages” secrete a variety of growth factors, like *EGF*, *FGF* and *VEGF*, and immunosuppressive factors including *IL-10*, *PGE_2_* and *TGF-*β, thus providing a tumor-friendly micromilieu concomitant with a much worse prognosis for the afflicted patients [41,43,44,45]. However, whether LPS (or more likely endogenous *TLR4* ligands) will cause a rather acute inflammatory tumor micromilieu remains unclear. As shown here, induction of pro-inflammatory cytokines was solely induced in hybrid cells, but not parental cells. Thus, more breast cancer cells as well as breast cancer × normal cell hybrids have to be analyzed first. Conjointly, the role of *IL-6* in breast cancer has to be further clarified since recent studies revealed that *IL-6* appears to play a critical role in the growth and metastasis of breast cancer cells, renewal of breast cancer stem cells and drug resistance of breast cancer stem cells [46]. Thus, activation of *TLR4* signaling by LPS or endogenous ligands may foster the renewal and expansion of breast cancer stem cells. It would also be worthwhile to speculate about the role of *TNF-*α, which is not only a pro-inflammatory cytokine, but which has also been determined as a pro-fusogenic factor [47,48,49]. For instance, we have recently demonstrated that the fusion of M13SV1 breast epithelial cells and MDA-MB-231 and MDA-MB-435 breast cancer cells is promoted by *TNF-*α [47]. Thus, secretion of *TNF-*α either induced by LPS or endogenous ligands could promote the fusion of tumor cells and other cells. 

In accordance to the differential expression of LPS and CpG-ODN target genes both compounds also induced a differential *AKT* and *ERK1/2* signaling in the cells. Interestingly, both parental cells showed a similar *ERK1/2* activation pattern, with a peak maximum after 2, 6 and 48 h of CpG-ODN and LPS stimulation, whereas in M13MDA435-3 hybrid cells the peak maximum of *ERK1/2* phosphorylation was detected after 6 h and 48 h. Conjointly, *AKT* S473 and *AKT* T308 phosphorylation induced by CpG and LPS stimulation was solely detected in M13MDA435-1 and -3 hybrid cells, but not parental cells. It is well recognized that *AKT* is a central signaling node being involved in various cellular functions including metabolism, proliferation and survival [38]. Western blot data demonstrated that LPS induced *AKT* phosphorylation both at position S473 and T308 (Figure 5B), representing the two activation sites of *AKT* [38]. Thus, stimulation of M13MDA435-1 and -3 hybrid cells with LPS should result in full *AKT* activation concomitant with the induction of *AKT* mediated cellular processes including survival [38]. However, as shown recently, LPS potently induced apoptosis in M13MDA435-1 and -3 hybrid cells [33]. It can thus be concluded that two opposing signal transduction pathways regulating cell survival are engaged by LPS in M13MDA435-1 and -3 hybrid cells. This would also apply to the transcription factor *NF-*κ*B*. On the one hand, LPS induced *TLR4* signaling leads to nuclear translocation of *NF-*κ*B* concomitant with *IFN-*β expression and induction of apoptosis [33]. These data are in view with findings of Jung and colleagues demonstrating that the LPS induced apoptosis of cultured microglia cells was not only dependent on *IFN-*β, but was also dependent on *NF-*κ*B* activation mediating NO synthesis (via inducible NO synthase (iNOS) induction, *caspase-11* induction and its subsequent activation [50]. On the contrary, survival of endothelial cells is mediated by *TNF-*α in a *NF-*κ*B* dependent mechanism, whereby constitutive or inducible *NF-*κ*B*-independent pathway(s) protects HUVECs from cell death [51]. Likewise, a link between *Bcl-2* and *NF-*κ*B* signaling and suppression of apoptosis in ventricular myocytes has been reported [52], suggesting that *NF-*κ*B* plays a dual role in both inducing and preventing apoptosis. The finding that LPS potently induced apoptosis in M13MDA435-1 and -3 hybrid cells albeit pro-survival pathways are simultaneously engaged might be thus attributed to a differential kinetics and strength of the different *TLR4* induced pathways. In M13MDA435-1 and -3 hybrid cells activation of *AKT* (and possibly *NF-*κ*B*) may thus not be strong enough to counteract LPS induced pro-apoptotic pathways, thus preventing cell death. 

Whether the differential *AKT* activation in M13MDA435-1 and -3 hybrids in comparison to the parental cells might also be attributed to differential receptor expression levels remains to be elucidated. As shown in Figure 1A M13SV1-EGFP-Neo human breast epithelial cells expressed markedly lower levels of *TLR4*, *TLR9*, *TRIF*, *Myd88* and *TRAF6* suggesting that rather weak *AKT* T308 levels as well as the lower induction of target gene expression in response to LPS and CpG (Figure 2 and Figure 3) might be attributed to the cells overall lower receptor and signaling protein expression. However, markedly enhanced *pAKT* S473 levels were detected in LPS and CpG treated M13SV1-EGFP-Neo cells indicating that despite lower *TLR4* and *TLR9* expression levels activation of *AKT* at position S473 is potently induced. Moreover, *TLR4* and *TLR9* as well as *TRIF*, *Myd88* and *TRAF6* expression levels of MDA-MB-435-Hyg breast cancer cells were comparable to M13MDA435-1 and -3 hybrid cells. Nonetheless, rather weak *AKT* S473 to rather low *AKT* T308 levels were observed in LPS and CpG treated MDA-MB-435-Hyg cells, revealing that the activation of signal transduction cascades does not only depend on the expression levels of receptors and signal transduction proteins, but most likely also to other regulatory mechanisms.

Cell migration studies revealed that the locomotory activities of the parental cell lines and hybrid cell clones were significantly blocked by LPS and CpG-ODN, which is contrary to findings revealing that both LPS and CpG-ODN could promote tumor cell invasion and even metastasis formation [13,14,15,18,19,20,53]. On the contrary, other studies pointed out that e.g., silencing of *TLR4* increased tumor progression and lung metastases in a murine model of breast cancer [54] and that *TLR9* agonists could induce apoptosis in A20 lymphoma cells [55] and neuroblastoma cells [56]. Because LPS potently induced apoptosis in M13MDA435-1 and -3 hybrid, we assume that the LPS-impaired migratory behavior of the cells was rather attributed to an increased number of apoptotic cells in the assay. However, it cannot be ruled out completely that LPS might induce signal transduction pathways and/or might cause an altered gene expression profile that ultimately impairs the migratory activity of the cells. This assumption would be in line with the finding that the locomotory activity of MDA-MB-435-Hyg breast cancer cells was significantly impaired within the presence of 150 ng/mL LPS. As published recently, the viability of MDA-MB-435-Hyg was not affected by LPS [33]. Because the cell viability was also not affected by CpG-ODN, we conclude that the *TLR9* dependent inhibition of cell migration was as well attributed to intracellular signaling and/or an altered gene expression profile caused by CpG-ODN induced *TLR9* signaling. In fact, various data revealed that *TLR* agonists could inhibit chemotaxis of monocytes through inhibition of receptor expression [57] or endocytic clearance of receptors from the plasma membrane [58]. Additionally, *TLR* signaling could impair monocyte chemotaxis independent of receptor downmodulation through synergized effects of p38 MAPK and global *Rap-1* activation, which ultimately abolished actin reorganization [59]. Whether a similar mechanism might be responsible for the CpG-ODN dependent inhibition of the migration of the investigated cells remains to be elucidated.

## 4. Materials and Methods

### 4.1. Cell Culture

All cell lines were cultivated at 37 °C and 5% CO_2_ in a humidified atmosphere as recently described [25,26,33]. Human M13SV1-EGFP-Neo breast epithelial cells exhibiting stem cell properties were derived from human M13SV1 cells (kindly provided by James E. Trosko, Michigan State University, East Lansing, MI, USA [60]) by stable transfection with the pEGFP-Neo plasmid [25]. Cells were cultivated in MSU-I basal media (Biochrom GmbH, Berlin, Germany) supplemented with 10% fetal calf serum (FCS) (Biochrom GmbH, Berlin, Germany), 1% Penicillin/Streptomycin (100 U/mL Penicillin, 0.1 mg/mL Streptomycin; Sigma-Aldrich, Taufkirchen, Germany), 10 µg/mL human recombinant *EGF*, 5 µg/mL human recombinant *Insulin*, 0.5 µg/mL Hydrocortisone, 4 µg/mL human *Transferrin*, 10 nM β-Estrogen (all reagents were purchased from Sigma Aldrich, Taufkirchen, Germany), and 400 µg/mL G418 (Biochrom GmbH, Berlin, Germany). MDA-MB-435-Hyg human breast cancer cells were derived from MDA-MB-435 cells (HTB 129; LGC Standards GmbH, Wesel, Germany) by stable transfection with the pKS-Hyg vector. Cells were cultured in DMEM (Sigma Aldrich, Taufkirchen, Germany) supplemented with 10% FCS (Biochrom AG, Berlin, Germany), 1% Penicillin/Streptomycin (Sigma Aldrich, Taufkirchen, Germany) and 200 µg/mL Hygromycin B (Pan-Biotech, Aidenbach, Germany). M13MDA435-1 and -3 hybrid cell clones were derived from spontaneous fusion events between M13SV1-EGFP-Neo cells and MDA-MB-435-Hyg cells [25,32]. Hybrid cells were cultured in DMEM (Sigma Aldrich, Taufkirchen, Germany) supplemented with 10% FCS (Biochrom GmbH, Berlin, Germany), 1% Penicillin/Streptomycin (Sigma Aldrich, Taufkirchen, Germany), 400 µg/mL G418 (Biochrom GmbH, Berlin, Germany) and 200 µg/mL Hygromycin B (Pan-Biotech, Aidenbach, Germany).

### 4.2. RT-PCR

Total RNA was isolated from cells (1 × 10^6^) by using the NucleoSpinR^®^ RNA Kit II (Macherey-Nagel GmbH, Düren, Germany) in accordance to the manufacturer’s instructions. cDNA was reverse transcribed from mRNA using the RevertAid™ First Strand cDNA Synthesis Kit (VWR International, Darmstadt, Germany) are referred to instruction manual. For PCR (total volume 25 µL per reaction) 1.25 U Taq Polymerase, 1× reaction buffer, 2 mM MgCl_2_, 2 µM dNTPs (all reagents from VWR International, Darmstadt, Germany) and 100 µM primers (Life Technologies, Darmstadt, Germany) were used. The cycling conditions comprised of an initial denaturation step (5 min at 95 °C), 35 cycles of amplification (30 s 94 °C; 30 s appropriate annealing temperature; 30 s 72 °C) and final elongation (10 min 72 °C). PCR products were separated on a 1% TAE agarose gel. PCR bands were visualized by GelRed™ staining (VWR International GmbH, Darmstadt, Germany) and the GelDoc^TM^ EZ Imager System (Bio-Rad, Munich, Germany). Primer pairs used in this study are summarized in Table 1.

### 4.3. Extraction of Nuclear Proteins

Harvested cells (2 × 10^6^) were resuspended in culture media and were treated with 100 ng/mL LPS (Sigma-Aldrich, Taufkirchen, Germany) or 100 ng/mL CpG-ODN (Invivogen, San Diego, CA, USA), respectively, for 2 h at 37 °C and 5% CO_2_ in a humidified atmosphere. Non-stimulated cells served as a control. The NE-PER Nuclear and Cytoplasmic Extraction Reagent Kit (Thermo Fischer Scientific, Bonn, Germany) was used for purification of *NF-*κ*B* and *IRF-7* from nuclear extracts in accordance to the manufacturer’s instructions. Nuclear extracts were boiled in 3× Laemmli Sample Buffer (5 min, 95 °C) [61] and stored at −80 °C prior to SDS-PAGE and Western Blot analysis.

### 4.4. Western Blot Analysis

Cells (2 × 10^5^) were seeded in 6-well plates and were stimulated with either 100 ng/mL LPS (Sigma-Aldrich, Taufkirchen, Germany) or 100 ng/mL CpG-ODN (Invivogen, San Diego, CA, USA), respectively, for 2, 4, 6, 12, 24, and 48 h at 37 °C and 5% CO_2_ in a humidified atmosphere. Subsequently, cells were harvested and lysed in 3 × Laemmli Sample Buffer (5 min, 95 °C) [61]. Samples were separated on a 10% or 12%, respectively, SDS-PAGE and transferred to an Immobilon PVDF nitrocellulose membrane (EMD Millipore, Darmstadt, Germany) under semi-dry conditions. Membranes were blocked with 10% (*w*/*v*) not-fat milk powder or 5% BSA in TBS-T. Bands were visualized using the Pierce ECL Western Blotting Substrate (Thermo Fischer Scientific, Bonn, Germany) in accordance to the manufacturer’s instruction and the Aequoria Macroscopic Imaging System (Hamamatsu Photonics Germany, Herrsching am Ammersee, Germany). Antibodies used for Western blot analysis are listed in Table 2.

### 4.5. 3D-Collagen Matrix Migration Assay

The migratory behavior of the cells in response to LPS and CpG-ODN was performed by using the 3D-collagen matrix migration assay as described recently [24,25,26,62,63,64]. Briefly, cells (4 × 10^4^ to 6 × 10^4^) were mixed with liquid collagen solution (Purecol; Nutacon BV, Leimuiden, The Netherlands) was mixed with 10× MEM (Sigma-Aldrich, Taufkirchen, Germany), and 7.5% sodium bicarbonate solution (Sigma-Aldrich, Taufkirchen, Germany). In dependence of the experimental setting different concentrations of either LPS or CpG-ODN were added. The cell × collagen mixture was filled in self-constructed cell migration chambers and the collagen was allowed to polymerize. Subsequently, cell migration chambers were placed under a microscope in a compartment tempered to 37 °C. The migration of the cells was recorded by time-lapse video microscopy overnight (at least 15 h). To analyze the migration of the cells, in each experiment 30 cells were randomly selected and the paths of the cells were tracked in 15 min real-time intervals using a manual cell tracking software application. 

### 4.6. Statistical Analysis

Statistical significance of the cell migration data was calculated using the Mann-Whitney *U*-test. 

## 5. Conclusions

In summary, here we have shown that M13MDA435-1 and -3 hybrid cells derived from MDA-MB-435-Hyg human breast cancer cells and M13SV1-EGFP-Neo breast epithelial cells exhibit a differential *TLR4* and *TLR9* signaling, which is in view with the cell fusion hypothesis that hybrid cells could exhibit novel properties. Cell fusion is a random and unpredictable process that is chiefly attributed to HST representing the merging of the parental nuclei—a process which is characterized by loss of whole chromosomes, unequal and random segregation of chromosomes to the daughter cells and even chromothripsis [28,29,65,66,67]. The random distribution of parental chromosomes to daughter cells was recently visualized by Zhou and colleagues, which further showed that hybrid cells exhibited a greater extent of DNA double strand breaks [66]. Thus, cell fusion and HST might be associated with chromothripsis specifying the shattering and random rearrangement of one or more chromosomes [67]. Chromothripsis is a common phenomenon in many (if not all) cancers and has been associated with the loss of tumor suppressors, dysregulation of genes with known cancer links and oncogene amplification [67]. Moreover, chromosome segments that fail to get reincorporated can circularize to become double minutes, which are frequently amplified [67]. Karyotypes of hybrid cells derived from the fusion of hamster cells and human tumor cells revealed the existence of a series of small unidentifiable chromosomes/chromosomal structures [68] that may have originated from chromothripsis. 

Consequently, cell fusion is a potent mechanism that gives rise to unique hybrid cells that, concomitant with their progenies, will increase the heterogeneity of the tumor mass. How these cells ultimately behave in the tumor microenvironment and react to surrounding stimuli strongly depends on the cells receptor repertoire and kinetics of signal transduction cascade controlled by the cells’ genetic and epigenetic background concomitant. 

## Figures and Tables

**Table 1:** Used primer sequences for PCR.

Target	Primer Sequence 5′-3′	Annealing Tempeature °C	Amplicon (bp)
*IFN-*β	F: AGTAGGCGACACTGTTCGTG	60	174
	R: AGCCTCCCATTCAATTGCCA		
*IL-1*β	F ^1^: AGCCATGGCAGAAGTACCTG	54	219
	R: TCCATGGCCACAACAACTGA		
*IL-6*	F: ATGAACTCCTTCTCCACAAGCGC	60	628
	R: GAAGAGCCCTCAGGCTGGACTG		
*MCP-1*	F: CCCCAGTCACCTGCTGTTAT	60	135
	R: AGATCTCCTTGGCCACAATG		
*TNF-*α	F: AACATCCAACCTTCCCAAACG	54	109
	R: GACCCTAAGCCCCCAATTCTC		
*TRAIL*	F: GAGCTGAAGCAGATGCAGGAC	60	137
	R: TGAGGAGTTGCCACTTGACT		
β*-actin*	F: GTGACGTTGACATCCGTAAAGACC	55	290
	R: TCAGTAACAGTCCGCCTAGAAGCA		

^1^ F = Forward Primer, R = Reverse Primer.

**Table 2:** Antibodies used.

Antibody	Manufacturer
*AKT*, rabbit monoclonal	Cell Signaling ^1^
*pAKT* S473, rabbit monoclonal	Cell Signaling ^1^
*pAKT* T308, rabbit monoclonal	Cell Signaling ^1^
*BAX*, rabbit monoclonal	Cell Signaling ^1^
*BCL-2*, mouse monoclonal	Cell Signaling ^1^
*ERK1/2*, rabbit polyclonal	Cell Signaling ^1^
*pERK1/2*, rabbit polyclonal	Cell Signaling ^1^
*Histone H3*, rabbit polyclonal	Abcam ^2^
*IFN-*β, mouse monoclonal	Biozol ^3^
*IL-1*β, rabbit monoclonal	Cell Signaling ^1^
*IL-6*, rabbit monoclonal	Cell Signaling ^1^
*IRAK1*, mouse monoclonal	Abgent Inc. ^4^
*IRF7*, rabbit polyclonal	Cell Signaling ^1^
*MCP-1*, rabbit polyclonal	Cell Signaling ^1^
*Myd88*, rabbit monoclonal	Cell Signaling ^1^
*NF-κB* p65, rabbit polyclonal	Santa Cruz Biotech ^5^
*TLR4*, rabbit polyclonal	Santa Cruz Biotech ^5^
*TLR9*, rabbit polyclonal	ProSci Inc. ^6^
*TNF-*α, rabbit monoclonal	Cell Signaling ^1^
*TRAIL*, rabbit monoclonal	Cell Signaling ^1^
*TRAF6*, rabbit monoclonal	Cell Signaling ^1^
*TRIF*, rabbit polyclonal	Cell Signaling ^1^
*elf4E*, rabbit monoclonal	Cell Signaling ^1^
β*-actin*, rabbit monoclonal	Cell Signaling ^1^
anti-mouse-IgG-HRP-linked	Cell Signaling ^1^
anti-rabbit-IgG-HRP-linked	Cell Signaling ^1^

^1^ New England Biolabs GmbH, Frankfurt am Main, Germany; ^2^ Abcam, Cambridge, UK; ^3^ Biozol Diagnostica Vertrieb GmbH, Eching, Germany; ^4^ Abgent Inc., San Diego, CA, USA; ^5^ Santa Cruz Biotech, Heidelberg, Germany; ^6^ ProSci Inc., Poway, CA, USA.

## Abbreviations

CpG-ODN	CpG-oligodeoxynucleotide
DAMPs	danger-associated recognition patterns
HST	heterokaryon-to-synkaryon transition
lncRNA	long non-coding RNA
LPS	lipopolysaccharide
miRNA	microRNA
mTORC2	mechanistic target of Rapamycin complex 2
PAMPs	pathogen-associated recognition patterns
*PDK1*	phosphoinositide-dependent kinase-1
*PI3K*	phosphatidylinositol 3-kinase
PIP3	phosphatidylinositol-3,4,5-triphosphate
*TLRs*	toll-like receptors
